# The Perceived Benefits and Self-Efficacy of an Exercise Intervention on Tobacco Withdrawal Symptoms: A Qualitative Study Based on the Health Belief Model

**DOI:** 10.21315/mjms2024.31.3.15

**Published:** 2024-06-27

**Authors:** Ruslan Nur-Hasanah, Yasin Siti Munira, Mohd Nasir Nadzimah, Isa Mohamad Rodi

**Affiliations:** 1 Faculty of Sports Science and Recreation, Universiti Teknologi MARA, Shah Alam, Selangor, Malaysia; 2 Department of Public Health Medicine, Faculty of Medicine, Universiti Teknologi MARA, Sungai Buloh, Selangor, Malaysia; 3 Department of Pathology, Faculty of Medicine, Universiti Teknologi MARA, Sungai Buloh, Selangor, Malaysia

**Keywords:** exercise intervention, perceived benefit, self-efficacy, smoking cessation, qualitative study, health belief model

## Abstract

**Background:**

This study examined the effectiveness of an exercise programme as an adjunct to smoking cessation treatments. The effects of exercise on smoking habits and tobacco withdrawal symptoms (TWS) were evaluated among smokers who were in the pre-contemplation and contemplation stages.

**Methods:**

This was a case study with convenience sampling techniques. This study lasted approximately 2 years, beginning in February 2016. This study was divided into two phases: an intervention phase followed by an interview. The participants were invited to undergo the 8-week supervised moderate aerobic exercise programme. This qualitative study involved 14 participants selected from the intervention phase. They were interviewed about their experiences using a semi-structured questionnaire guided by the health belief model.

**Results:**

This study involved 14 participants who were aged between 26 years old and 40 years old and smoked from 11 to 20 cigarettes per day. Most participants perceived benefits and self-efficacy regarding smoking habits and tobacco withdrawal symptoms (TWS) following the exercise intervention.

**Conclusion:**

This study demonstrated that moderate exercise might be helpful in increasing self-efficacy in smoking cessation and the findings encourage further research on exercise programmes as an adjunct to smoking cessation treatments in Malaysia.

## Introduction

Smoking is a substantial threat to public health. However, the smoking cessation rate remains low worldwide (1). Quitting smoking is difficult for most smokers. Almost two-thirds of smokers attempt to quit, but only a small percentage of 5% to 15% eventually succeed in quitting (2). In Malaysia, 48.9% of current smokers attempted to quit in the past 12 months (3). However, a successful smoking cessation rate of only 2%–6% has been reported in Malaysia (4). The success rate of smoking cessation if influenced by several factors, such as sociodemographic factors, smoking variables and smoking cessation treatment (1).

There have been clinical trials on smoking cessation treatments such as behavioural counselling, medication, combination therapy and nicotine replacement therapy (NRT). However, there is still insufficient evidence for particular treatments (5, 6) due to several sociodemographic factors (7) that significantly influence the attributes for successful smoking cessation (8). Indeed, demographic characteristics still exhibit disparities and clinical treatment is still needed for success in smoking cessation (1).

Several new adjunct treatments are being introduced as part of continuous efforts to promote newer strategies for current smoking cessation treatment (9). Even adjunct treatment does not promise successful quitting, but it may motivate behavioural change to discourage routine smoking (10). Indeed, the combination of pharmacotherapy and non-pharmacotherapy treatment is still most effective (6). Thus, the study of non-pharmacological therapy that shows high efficiency in smoking cessation is also important to increase the treatment efficiency of combination therapy.

Exercise has been used as an adjunct treatment to reduce addiction in animal and human studies (11). A systematic review also found that exercise is a useful aid in tobacco control (12) because it mimics the effects of smoking. Both have similar physiological immediate effects, such as increased blood pressure, heart rate and adrenaline levels (13). Exercise also produces calmness effects due to beta-endorphin release, which regulates mood and provides euphoria effects (14, 15). Thus, this might explain why exercise has been used as an adjunct treatment to reduce cravings and withdrawal symptoms in previous studies (16). Thus, the study of the effects of exercise on tobacco withdrawal symptoms (TWS) warrants further investigation (12).

Several theories and models have been used in smoking cessation intervention strategies. The health belief model (HBM) posits that individuals will act to improve their health if they believe they have the capability to do so. The HBM has two key beliefs: perceived susceptibility to illness and anticipation of its severity. These key beliefs consist of two evaluations: the perceived benefit and efficacy of recommended health behaviour and the addition of a cue of action (17). The HBM emphasises the role of individual beliefs and perceptions in shaping health behaviours (18). Addressing the effectiveness of exercise and self-efficacy based on the HBM can motivate individuals to act towards quitting smoking. Additionally, integrating exercise into smoking cessation interventions aligns with the principles of the HBM by providing individuals with tangible strategies to reduce their dependence on smoking and act as a coping mechanism. In the present study, we examined the effectiveness of an exercise programme using in-depth interviews to explore each participant’s subjective experiences.

## Methods

### Study Design and Sampling Method

**Figure 1:**
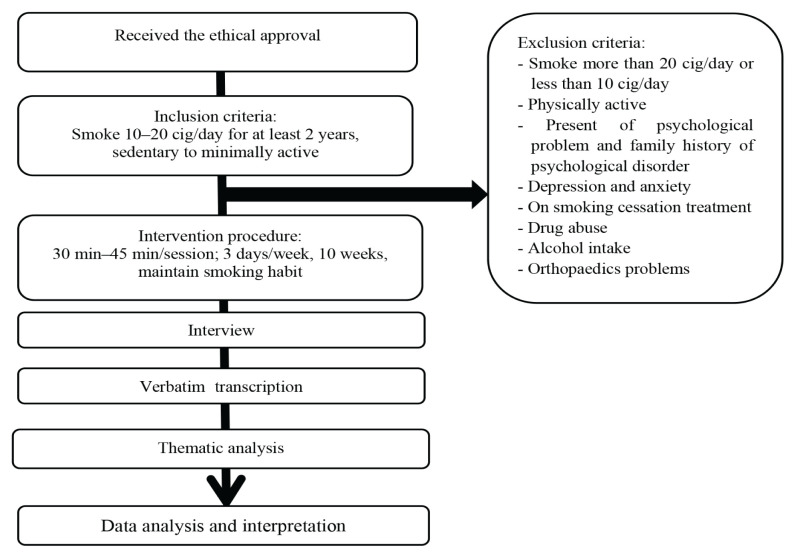
Summary of study flow

The flow chart of this study is shown in Figure 1. This was a qualitative study with a case study design because the participants were bound to an 8-week exercise intervention programme (19) with the case study exploring the phenomenon at a specific point in time. This study used a convenience sampling method.

### Sample Size

The sample size for the case study ranged from 1 to 95, whereas the typical sample size for the qualitative study ranged from 10 to 50 samples. Since this study was conducted based on the HBM constructs, theoretical saturation was achieved after all the sample codes had been observed at least once (21). Data collection was discontinued when all the themes had sample codes for their subthemes.

### Participant Recruitment

Participants were recruited using posters, emails and signs. Recruitment was performed from February 2016 until 2018.

### Screening Procedures

The screening procedures involved screening the participants’ physical activity level, smoking status, physical readiness, health status and mental illness.

### Intervention

Eligible participants underwent the exercise intervention for 8 weeks. The training was conducted in a gymnasium located at Level 7, Academic Building, Universiti Teknologi MARA Sg. Buloh, Selangor, Malaysia.

### Participant Characteristics

Participants were recruited from a government health institution around Sg. Buloh, Selangor. Participants were screened to ensure that they met our inclusion criteria: males over 20 years–45 years of age with no chronic disease, who smoked 10–20 cigarettes per day, were minimally active and sedentary and did not have anxiety or depression.

### Question Piloting

The questions for the semi-structured interview were designed based on the health belief model by three panels. Two panels determined the suitability of questions for each of the HBM constructs. After a series of modifications, the prefinal version was produced and was piloted among four smokers. Any ambiguity regarding the questionnaire was corrected and the questionnaire was repiloted among other participants.

### Instrument

This study used a semi-structured interview questionnaire that consisted of 10 questions to represent the five constructs of the HBM, as shown in Table 1.

### Site and Equipment of the Interview Session

The interview was conducted at numerous places in a comfortable and air-conditioned room in a one-on-one interview setting. The interview was performed using several recording devices (Android, Samsung Jpro), the questionnaire, participants’ forms and consent forms.

### Data Collection and Data Analysis

The participants who underwent the exercise intervention programme were interviewed with audio recordings. The interview was conducted approximately 16 min–35 min in Malay and mixed with English terminology. The audio recordings were subjected to verbatim transcription. The verbatim transcriptions were performed by five experienced freelancers. The text was entered into Microsoft Word 2016 version 16.0 documents and was used for the thematic analysis. For thematic analysis, the transcriptions were imported into ATLAS Ti version 8.0. Then, the quotes were coded with open coding based on the five constructs in the HBM. The code was also tagged into several subthemes. Then, the data were classified and summarised.

### Validity of the Study

The study’s validity was based on two methods: member checking and peer debriefing. The reflexivity process involved reflecting on the construct related to the understanding of social reality. The researcher used the smokers’ experience and knowledge gained through observation when commenting on and interpreting the responses (19).

## Results

### Demographic Characteristics

The study participants’ sociodemographic characteristics are shown in Table 2.

### Qualitative Findings

This paper focus only on two results which are perceived benefit and self-efficacy.

#### Perceived Benefit

##### Improved smoking habits

Some participants claimed that the exercise intervention programme improved their smoking habits by reducing their smoking activity. All the responses showed that several smokers noticed changes in their smoking habits during the exercise intervention period.

*Other than that, the changes can also be seen through my smoking habit (P1, 36).*

*During the training programme, while exercising on the treadmill, I noticed that I had reduced my smoking (P4, 32).*

##### Prolonged abstinence period

The participants reported that regular exercise reduced the urge and craving to smoke. The participants even claimed that they could control their urges and cravings when they were exercising.

*When I wanted to start the exercise session, I did not smoke for approximately two hours before. It will take some time because of the exercise. For me, the longest gap between smoking a cigarette and exercising would be about two to three hours (P3, 39).*

*The gap between waking up and smoking is* *increasing (P8, 29).*

##### Reduced urges and cravings

The participants’ experience of a reduction in the difficulty of abstinence indicated a reduction in TWS. They noticed that the severity of TWS before the exercise intervention affected the strength of the desire to smoke and the physical symptoms when abstaining from smoking.

*When I was tired because of the exercise, I found that my desire to smoke had decreased. The exercise’s effects are that my body feels lighter than before, and the urge to smoke can also be controlled. Now, I truly believe that exercise can reduce the urge to smoke (P8, 29).*

*Yes, even though I still have not quit, reduced my habit, and at least now I know that when I exercise, I can reduce the urge to smoke (P11, 32).*

##### Reduced severity of tobacco withdrawal symptoms

The participants reported experiencing a reduction in the severity of TWS during abstinence after the exercise intervention programme. The reduction was noticed before the exercise intervention, when the desire to smoke and physical symptoms were felt less often.

*Before the exercise programme, the time when I had to abstain from smoking was so difficult. My smoking has been reduced. The desire is barely there, but it has been reduced (P11, 32).*

*I feel that my smoking is slightly reduced. Before this, my hand would shake when I had to abstain from smoking, so that is the effect... after the exercise (P13, 35).*

##### Reduced number of cigarettes

The participants noted that exercise reduced the number of cigarettes smoked on the days they exercised. They also found that the number of cigarettes was consistently reduced after the intervention before returning to smoking a regular number of cigarettes, similar to before the exercise intervention.

*A day before, I would reduce my cigarettes to about one or two (P3, 39).*

*During the exercise programme, I had less urge to smoke and reduced the number of cigarettes smoked compared to before the programme (P8, 29).*

#### Self-efficacy

Several participants believed that the exercise intervention could help in smoking cessation because they experienced a change before and after being engaged in the exercise intervention. The majority of the participants believed in the benefits of exercise and that exercise can be used as an aid in smoking cessation.

##### Belief that exercise can help

Several participants believed that the exercise intervention could help with smoking. Several participants agreed that exercise could help reduce the desire to smoke and the number of cigarettes smoked per day. Participants also noted that exercise was not effective for all smokers but agreed that it distracted them from thinking about a cigarette.

*When exercising, it is’ okay to delay smoking and let it be for a while. It is’ possible to reduce smoking; I am sure the programme helps to reduce it …but only for some people…For me, it can help (P8, 29).*

*When I am active, it is’ possible for me to not think too much about smoking. So, I think it is’ possible., exercise does help to reduce this habit if the person is consistent (P14, 31).*

##### Exercise as an aid

Several participants believed that exercise possibly aided in smoking cessation but perceived that exercise could not directly cause them to stop; they experienced only several changes. Exercise was considered an aid because they found that it helped distract them from thinking about smoking. They also felt that exercise could be an aid because it improved their fitness and made them feel healthy.

*Yes, it is’ possible that exercise can help someone quit smoking... Because I feel different during the programme… I was able to control it… I could ignore the urge; I felt like I wanted to smoke, but I was able to think ‘it is okay, put it on hold first’ (P4, 32).*

*Yes, it is possible. I think it is one type of help….Aaa…mmm, I mean exercise, such as activities. This programme promotes exercise, and smokers think less about cigarettes… my body does not feel like it needs cigarettes (P22, 30).*

*I think it is possible for exercise to become an aid ….Hmm, if one’s determination is strong enough and if the exercise is done continuously, the possibility exists within ourselves (P28, 25).*

## Discussion

The effectiveness of a particular health intervention is not only influenced by the physiological aspect of health but also by the behavioural aspect in terms of emotion and health decision-making (22). Thus, this study found several important aspects related to the likelihood of engaging in exercise for smoking cessation. This study focused on perceived benefit and self-efficacy because they are considered the most powerful constructs influencing the effectiveness of exercise, especially in reducing TWS.

The participants reported that the exercise intervention benefited them in several aspects, such as improving several smoking variables and their quality of life. Specifically, the exercise intervention improved smoking variables such as reduced smoking habits, the number of cigarettes smoked, the desire to smoke, cravings, prolonged abstinence and reduced severity of TWS. In this context, the perceived benefit can also be the strongest predictor when establishing evidence for a positive intervention impact (23) that leads to a favourable outcome (24). However, several factors might affect the perceived benefits of exercise, such as self-efficacy (24) and improved energy and psychological variables (25). Moreover, the perceived benefit can be enhanced throughout the intervention period (30), as observed in this study and it affects the likelihood of exercising (targeted health behaviour) for smoking cessation purposes. The perceived benefit should outweigh the perceived barrier to warrant that the targeted health behaviour be performed (26) because of the reduced perceived barrier (27).

Most participants also noticed that the intervention prolonged the abstinence period and extended the period before subsequent cigarette consumption. A prolonged smoking abstinence period could reduce the craving or be intentionally undertaken based on participants’ personal experiences. Participants also noted that if they smoked and immediately exercised, they felt uncomfortable and experienced shortness of breath. Thus, to avoid these uncomfortable experiences during exercise, they intentionally had a gap between cigarette smoking and the exercise regimen. Theoretically, the discomfort felt after smoking is a normal physiological response because carbon monoxide has a higher oxygen affinity and reduces its binding with haemoglobin, which subsequently promotes the fatigue, dyspnoea or leg pain reported by smokers compared to non-smokers (28). If the participants notice the positive effects of the intervention, it can strengthen the perceived benefits and enhance the adherence to exercise interventions (29, 30) This perceived benefit can encourage ex-smokers to perceive exercise as a part of their changed behaviour. Then, this can be a strong predictor of a smoker’s ability to adopt healthy behaviours by enhancing self-efficacy to exercise during smoking cessation.

Perceived self-efficacy was found to be unique to tobacco addiction. Efficacy can influence the initial phase of addictive habits while maintaining abstinence. Several types of efficacy changes in addictive behaviour related to this study included treatment behaviour self-efficacy, control-self efficacy, and abstinence self-efficacy. This study focused more on treatment self-efficacy because several participants believed that the exercise intervention could be an aid to reducing TWS and smoking habits. The positive experiences during the exercise intervention may be related to the participants’ ability to master the therapeutic task (exercise). This finding was also reported by a previous study on disease intervention (31). Self-efficacy is caused by several factors and indirectly related to the intervention’s success (32). Based on this study, factors such as mastery experience, self-persuasion and affective state were observed. These factors affected the participants’ perceived self-efficacy, reflecting confidence in their capacity to perform new health behaviours.

This study’s findings supported the fact that self-efficacy is affected by positive experiences. Thus, most participants who had perceived benefits before, during and after the exercise intervention tended to perceive self-efficacy in exercise. The participants with high self-efficacy had a higher tendency to improve increase the perceived benefits and adopt healthy behaviours (33) due to their mastery experiences. The participants in this study underwent repeated bouts of exercise sessions. Most of them felt good and succeeded in completing the exercise sessions, even if they were sedentary participants. The other factor related to self-efficacy is performance accomplishments. In this study, the participants achieved efficacy through performance accomplishments when they completed the exercise sessions, which gradually became longer and harder. The completion of this progression in intensity influenced their capabilities and confidence. However, self-efficacy is dynamic and needs to be maintained to ensure prolonged engagement with a new health behaviour because it can be threatened by perceived barriers such as physical, environmental and personal factors (34).

Based on this study, a person with perceived confidence is more likely to engage in specific healthy behaviours to improve their health (24). Self-efficacy has also been associated with adhering to an exercise programme, which potentially reduces the perceived barrier (33). A low belief in the efficacy of a pre-exercise intervention might lead to no change in the dependency on tobacco use, as shown in other addictive substance studies. The significance of the study findings would definitely impact the self-efficacy of the exercise intervention. The positive experience during exercise intervention, in addition to potentially increasing confidence, can also increase self-efficacy. Self-efficacy is important since low efficacy may be associated with dependency and substance abuse consumption (35).

In this study, most participants were confident and agreed that exercise helped reduce most of their TWS. This result is supported by a previous study, which reported that exercise can increase motivation and readiness among smokers in the precontemplation and contemplation stages (36). When participants have low efficacy beliefs about the treatment or therapy before an intervention, it can potentially contribute to an insufficient reduction in TWS. To have treatment efficacy with a particular aid or therapy, participants need to know the efficiency of treatment during cessation. However, if participants lack exposure to pharmacological and nonpharmacological aids, they may attempt to quit smoking without any assistance. The lack of knowledge about available treatments indicates that there is less publicity about smoking cessation treatments. This problem was reported in a previous study (37). This condition might increase the likelihood of unsuccessful quitting attempts.

Knowledge about the effect of available treatments is essential since it can increase efficacy, especially action efficacy (35). In this study, the participants might have increased their treatment beliefs because they engaged in exercise. In addition, exercise before cessation will build the confidence to quit smoking (36) or increase action self-efficacy. Unfortunately, this study did not aim for improvement beyond the contemplation stage. However, this study did observe a transient change from the precontemplation stage to the contemplation stage or vice versa. The pre-contemplation stage remained the same or transitioned into the contemplation stage. Perhaps when participants were in the contemplation stage, they might have kept postponing the action to quit (35) even though they perceived self-efficacy in the exercise intervention. However, it was too difficult to change to the action stage since this study was limited to only adjunct therapy. Consequently, exercise is essential because it can help in smoking cessation preparation to increase the smoker’s confidence to adopt new behaviour changes (36). Hence, this study showed that exercise may catalyse embracing a recent behaviour change (quit smoking). Therefore, exercise during cessation can help to reduce TWS, shift attention from smoking activity and provide coping strategies for negative moods. People who want to quit smoking can be more confident in using exercise as a coping strategy during their cessation.

## Conclusion

In conclusion, self-efficacy is one of the essential components of promoting exercise in smoking cessation. However, several types of self-efficacy exist at different stages of changes in smoking cessation. Indeed, determining the type of self-efficacy is important for predicting and explaining this study’s outcome. This study showed that participants also reported subjective experiences with the exercise intervention programme. Hence, the vital components of TWS (craving, urge and prolonged abstinence) and the number of cigarettes were improved during temporary smoking abstinence.

## Figures and Tables

**Table 1:** Semi-structured questionnaire

Constructs	Questions
Cue to action	Can you explain why did you start to exercise? In your opinion, what are the factors that encourage you to exercise?
Perceived benefit	Can you explain how does exercise can influence your smoking habit? Did the exercise intervention affect your smoking habit? Did the exercise training affect your desire to smoke when you had to abstain from smoking during the first, second and third measurement? Describe in detail your experience in general throughout the exercise intervention. How about your smoking habit after joining the exercise intervention programme?
Perceived threat	Can you explain, what are your expectations when joining this exercise intervention programme?
Self-efficacy	How can you be sure that exercise can influence you to overcome your smoking habit?
Perceived barrier	In your opinion, what are the factors that prevent you from exercising if you think exercise help to overcome your smoking habit?

**Table 2:** Participant characteristics

Sociodemographic characteristics	*n* (%)
Age (years old)	
18–25	1 (7.15)
26–40	13 (92.9)
Educational level	
Secondary school	4 (28.5)
Certificate	3 (21.5)
University	7 (50)
Cigarette per day	
1–10	4 (28.6)
11–20	10 (71.4)
